# Altered Temporal Dynamic Intrinsic Brain Activity in Late Blindness

**DOI:** 10.1155/2020/1913805

**Published:** 2020-06-20

**Authors:** Xin Huang, Zhi Wen, Chen-Xing Qi, Yan Tong, Han-Dong Dan, Bao-Jun Xie, Yin Shen

**Affiliations:** ^1^Eye Center, Renmin Hospital of Wuhan University, Wuhan, 430060 Hubei, China; ^2^Department of Radiology, Renmin Hospital of Wuhan University, Wuhan, 430060 Hubei, China; ^3^Medical Research Institute, Wuhan University, Wuhan, Hubei, China

## Abstract

Previous neuroimaging studies demonstrated that visual deprivation triggers significant crossmodal plasticity in the functional and structural architecture of the brain. However, prior neuroimaging studies focused on the static brain activity in blindness. It remains unknown whether alterations of dynamic intrinsic brain activity occur in late blindness (LB). This study investigated dynamic intrinsic brain activity changes in individuals with late blindness by assessing the dynamic amplitude of low-frequency fluctuations (dALFFs) using sliding-window analyses. Forty-one cases of late blindness (LB) (29 males and 12 females, mean age: 39.70 ± 12.66 years) and 48 sighted controls (SCs) (17 males and 31 females, mean age: 43.23 ± 13.40 years) closely matched in age, sex, and education level were enrolled in this study. The dALFF with sliding-window analyses was used to compare the difference in dynamic intrinsic brain activity between the two groups. Compared with SCs, individuals with LB exhibited significantly lower dALFF values in the bilateral lingual gyrus (LING)/calcarine (CAL) and left thalamus (THA). LB cases also showed considerably decreased dFC values between the bilateral LING/CAL and the left middle frontal gyrus (MFG) and between the left THA and the right LING/cerebelum_6 (CER) (two-tailed, voxel-level *P* < 0.01, Gaussian random field (GRF) correction, cluster-level *P* < 0.05). Our study demonstrated that LB individuals showed lower-temporal variability of dALFF in the visual cortices and thalamus, suggesting lower flexibility of visual thalamocortical activity, which might reflect impaired visual processing in LB individuals. These findings indicate that abnormal dynamic intrinsic brain activity might be involved in the neurophysiological mechanisms of LB.

## 1. Introduction

Visual deprivation leads to significant crossmodal plasticity in the brain's functional and structural architecture. Previous studies have demonstrated that visual deprivation triggers the visual cortex to other sensory processing, such as tactile [1], auditory [2], and olfactory functions [3], as well as higher cognitive functions (e.g., verbal memory [4] and episodic retrieval [5]). Furthermore, several neuroimaging studies have shown that blindness is associated with progressive atrophy of the visual pathway [6, 7] and visual cortices [8], as well as with abnormalities in non-visual areas [9, 10].

Recently, resting-state functional magnetic resonance imaging methods have been applied extensively to assess the effects of visual deprivation on the brain's functional architecture. Notably, visual deprivation has led to the reorganization of brain function. Liu et al. demonstrated that early blindness increases regional homogeneity (ReHo) in the visual cortex [11]. On their part, Jiang et al. reported that blindness increases regional spontaneous brain activity in visual areas and reduces it in sensorimotor and salience networks [12]. Visual deprivation has also been shown to induce significant abnormal interactions between the visual cortex and other sensory cortices. According to previous neuroimaging studies, blindness harbors abnormal interconnections between the visual cortex and other cortices: motor cortex [13, 14], Broca's area [15], and auditory cortex [16]. Striem-Amit et al. found that central V1 is more strongly connected to language areas, whereas peripheral V1 is more powerfully associated with spatial attention and control networks in blindness [17]. Per Wang et al., congenital blindness has increased network connectivity within the salience network and occipital cortex, as well as abnormal internetwork connectivity between the salience network and the frontoparietal networks and sensorimotor networks [18]. However, these resting-state fMRI studies focused on static intrinsic brain activity and connectivity and did not assess the temporal dynamic intrinsic brain activity in late blindness. Recently, neuroimaging studies have begun focussing on investigating dynamic brain activity or networks that can reflect information on the variability in the strength or spatial dynamic organization of the brain [19, 20]. Thus, we regard temporal dynamic brain activity analyses as a way to potentially deepen our understanding of brain activity changes in patients with blindness.

The human brain is a complex dynamic system capable of nonstationary neural activity and rapidly changing neural interaction. The human brain activity is inherently dynamic [21]. A map of the brain's dynamism reflects its temporal variability, which relates to the functional ability of neural networks [22]. Low-frequency oscillations (<0.08 Hz) of blood-oxygenation-level-dependent (BOLD) signaling in the human brain are physiologically meaningful. Notably, there is growing evidence that the temporal variability of BOLD signaling exists during the typical duration (a few minutes) of a resting-state scan of the human brain [23, 24]. The temporal variability of BOLD signaling plays a critical role in the implementation of various physiological functions, such as consciousness [25] and cognition [26]. Sliding-window analysis and clustering methods have been used to investigate temporal variability in BOLD signaling [27, 28].

To study changes in BOLD signaling over time, sliding-window correlation analysis, where the correlation is estimated for brain activity during multiple, possibly overlapping temporal segments (typically 30-60 s), has been widely deployed [29, 30]. The amplitude of low-frequency fluctuations (ALFF) method is a reliable and sensitive functional magnetic resonance imaging technology for the quantification of local intrinsic brain activity [31]. Recently, the dynamic ALFF (dALFF) with a sliding-window analysis was successfully used to investigate the temporal variability of brain activity in patients with generalized tonic-clonic seizures [32], poststroke aphasia [33], and schizophrenia [34]. However, it is largely unknown whether dynamic spontaneous brain activity changes occur in patients with blindness. Patients with retinitis pigmentosa (RP) offer a unique opportunity to study this issue. RP is an inherited retinal disease that primarily affects rod photoreceptor cells, followed by the degeneration of cone photoreceptor cells, eventually leading to blindness. Here, we selected RP patients who had experienced vision loss in adulthood. The goal of this study was to determine whether an altered dynamic spontaneous neural activity is present in blind patients. We hypothesized that blindness might be associated with abnormal dynamic spontaneous neural activity in vision and vision-related brain regions. Our findings may shed new light on the underlying pathological and compensatory mechanisms in blind patients.

## 2. Materials and Methods

### 2.1. Participants

Forty-one cases of late blindness (LB) (29 males and 12 females, mean age: 39.70 ± 12.66 years) and 48 sighted controls (SCs) (17 males and 31 females, mean age: 43.23 ± 13.40 years) participated in this study. All participants met the following criteria: (1) could be scanned with an MRI (e.g., no cardiac pacemaker or implanted metal devices); (2) did not have heart disease and claustrophobia; (3) did not have cerebral diseases (T1 images were checked by an experienced radiologist).

All LB subjects met the following criteria: (1) onset age of blindness >12 years; (2) had no ocular surgical history.

All SC subjects met the following criteria: (1) had no ophthalmic diseases (glaucoma, optic neuritis, retinal degeneration, etc.); (2) had visual acuity ≥1.0; (3) had no mental disorders.

Ethical statement: the study was approved by the medical research ethics committee and the institutional review board of the Renmin Hospital of Wuhan University Hospital. The protocol of the research followed the Declaration of Helsinki. All subjects provided written informed consent.

### 2.2. MRI Parameters

MRI scanning was performed on a 3-T magnetic resonance scanner (Discovery MR 750W system; GE Healthcare, Milwaukee, WI, USA) with eight-channel head coil. All subjects underwent MRI scanning (eight minutes) with eyes closed without falling asleep and 240 functional images were obtained. The more details on scanning parameters were showed in Table 1.

### 2.3. fMRI Data Processing

The fMRI data preprocessing was performed using Data Processing & Analysis of Brain Imaging toolbox (DPABI, http://www.rfmri.org/dpabi) [35], which is based on Statistical Parametric Mapping (SPM8) (http://www.fil.ion.ucl.ac.uk) implemented in MATLAB 2013a (MathWorks, Natick, MA, USA) and briefly the following steps [36]: (1) eliminate first ten time points for signal reaching equilibrium, and then slice timing and motion correction. For head motion parameters, more than 2 mm or for whom rotation exceeded 1.5°during scanning were excluded. (2) Individual 3D-BRAVO images were registered to the mean fMRI data [37]. (3) Covariates (six head motion parameters, mean framewise displacement (FD), global brain signal, and the average signal from white matter signal and cerebrospinal fluid) were used to regress out. (4) Linear trends were removed and filtered (0.01–0.08 Hz). Scrubbing regression was not performed because contiguous time points were necessary for dynamic analysis [38].

### 2.4. dALFF Variance Computing

A sliding-window approach was used to compute the dALFF using the Dynamic Brain Connectome (DynamicBC) toolbox (v2.0, http://www.restfmri.net/forum/DynamicBC) [39]. For the sliding-window approach, to avoid the introduction of spurious fluctuations, the minimum window length should be larger than 1/*f*min, where *f*min is the minimum frequency of the time series [40]. Here, a window length of 50 TR was considered as the optimal parameter to maintain the balance between capturing a rapidly shifting dynamic relationship and obtaining reliable estimates of the correlations between regions [41]. A window size of 50 TRs (100 s) and a window shifted by 10 TRs were selected [42]. Consequently, whole-length time courses were separated into 19 windows for each subject. An ALFF map was obtained for each sliding-window, and the ALFF of each voxel was standardized using *z*-transformation.

### 2.5. dFC Variance Computing

The altered dALFF brain regions were identified as regions of interest (ROIs). 6-mm radii around the B-LING/CAL [0, -72, 6] and L-THA [-6, -9, 6] coordinates were mapped as ROIs. A sliding-window approach via the DynamicBC toolbox (http://www.restfmri.net/forum/DynamicBC) was also used to obtain the whole-brain dFC maps of each seeded region. The variance of the time series of the correlation coefficient was estimated by calculating the standard deviation of *z* values at each voxel to assess dFC flexibility.

### 2.6. Clinical Evaluation

Clinical data, including age, sex, and disease duration were recorded.

### 2.7. Statistical Analysis

The chi-square (*χ*2) test and independent-sample *t* test were performed to assess the behavioral data between two groups using SPSS version 20.0 (SPSS Inc, Chicago, IL, USA) (*P* < 0.05 significant differences).

A one-sample *t* test was conducted to assess intragroup patterns of zdALFF maps using the DPABI software. A two-sample *t* test was used to assess zdALFF and the zdFC map difference between two groups' regressed covariates of age and sex and FD using the DPABI software. The Gaussian random field (GRF) method was used to correct for multiple comparisons (two-tailed, voxel-level *P* < 0.01, GRF correction, cluster-level *P* < 0.05).

Pearson correlation coefficient was used to assess the relationships between the dALFF and dFC values of different brain regions and clinical variables in the LB group using the SPSS version 20.0 software (SPSS Inc., Chicago, IL, USA).

### 2.8. Verification Analyses

To validate our dALFF findings, two different window lengths (30 TRs (60 s) and 100 TRs (200 s)) were calculated in the validation analysis. An ALFF map was obtained for each sliding window, and the dALFF of each voxel was standardized using z-transformation.

## 3. Results

### 3.1. Demographic Measurements

There are no significant differences in age between two groups There are significant differences in gender (*P* < 0.001) between two groups. The age of onset blindness is 22.56 ± 7.13 years in the LB group. Details are shown in Table 2.

### 3.2. Dynamic ALFF Variance Differences

**Figure 1:**
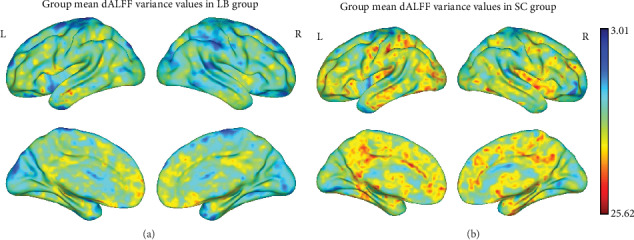
Spatial patterns of dALFF variance were observed at the group level in LB and SC groups in the typical frequency band (0.01–0.08 Hz). **W**ithin group mean dALFF variance maps within the LB (**a**) and SC (**b**). dALFF: dynamic amplitude of low-frequency fluctuation; LB: late blindness; SC: sighted controls; L: left; R: right.

**Figure 2:**
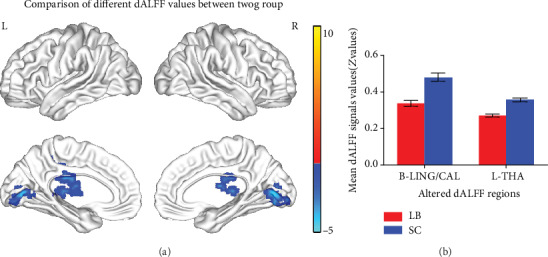
Comparison of different dALFF values between LB group and SC group. Significant dALFF values differences were observed in the B-LING/CAL, L-THA. The blue areas indicate lower dALFF values (voxel-level *P* < 0.01, GRF correction, cluster-level *P* < 0.05) (**a**). The mean values of altered dALFF values between the LB and SC groups (**b**). dALFF: dynamic amplitude of low-frequency fluctuation; LB: late blindness; SC: sighted controls; GRF: Gaussian random field; LING: lingual gyrus; CAL: calcarine; THA: thalamus; L: left; B: bilateral;.

The spatial distribution of dALFF maps between the two groups is shown in Figure 1. Compared with SCs, individuals with LB exhibited significantly lower dALFF values in the bilateral LING/CAL and left THA (Figure 2(a) (blue) and Table 3). The mean values of altered dALFF between the two groups are shown in Figure 2(b).

### 3.3. Dynamic FC Variance Differences

**Figure 3:**
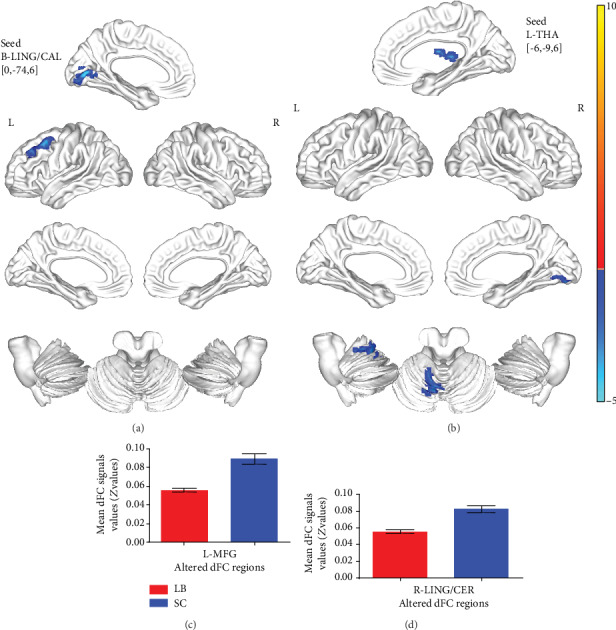
Comparison of different dFC values between LB group and SC group. Significant dFC values differences were observed in the L-MFG (**a**) and R-LING/CER (**b**). The blue areas indicate lower dFC values.(voxel-level *P* < 0.01, GRF correction, cluster-level *P* < 0.05) The mean values of altered dFC values between the LB and SC groups. (c, d). dFC: dynamic functional connectivity; LB: late blindness; SC: sighted controls; GRF: Gaussian random field; MFG: middle frontal gyrus; LING: lingual gyrus; CER: cerebelum_6; L: left; R: right.

Compared with SCs, LB cases exhibited markedly decreased dFC values between the bilateral LING/CAL and the left MFG and between the left THA and the right LING/CER (Figures 3(a) and 3(b) (blue) and Table 4). The mean values of altered dFC readings between the two groups are shown in Figures 3(c) and 3(d).

### 3.4. Receiver Operating Characteristic Curve

**Figure 4:**
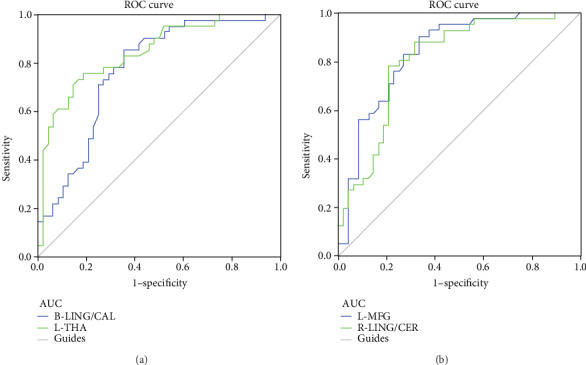
ROC curve analysis of the mean dALFF and dFC of altered brain regions. ROC curve in dALFF values: LB<HC, for B-LING/CAL, 0.770 (*P* < 0.001; 95% CI: 0.671–0.920); for L-THA, 0.843 (*P* < 0.001; 95% CI: 0.762–0.925) (**a**); ROC curve in dFC values: LB<HC, for L-MFG, 0.836 (*P* < 0.001; 95% CI: 0.752–0.920); for R-LING/CER, 0.806 (*P* < 0.001; 95% CI: 0.714–0.898) (**b**). ROC: receiver operating characteristic; dALFF: amplitude of low-frequency fluctuation; AUC: area under the curve; LING: lingual gyrus; CAL: calcarine; THA: thalamus; MFG: middle frontal gyrus; CER: cerebelum_6; L: left; R: right; B: bilateral;.

To test the sensitivity and specificity of dALFF and dFC value differences between the two groups, the areas under the ROC curve for dALFF were LB<HC, for bilateral LING/CAL, 0.770 (*P* < 0.001; 95% CI: 0.671–0.920); for left THA, 0.843 (*P* < 0.001; 95% CI: 0.762–0.925); (Figure 4(a)). The areas under the ROC curve for dFC were LB<HC, for left MFG, 0.836 (*P* < 0.001; 95% CI: 0.752–0.920); for right LING/CER, 0.806 (*P* < 0.001; 95% CI: 0.714–0.898); (Figure 4(b)).

### 3.5. Verification Analyses

In the verification analyses, we found that the group differences in dALFF variability with different window lengths (30 TRs (60 s) and 100 TRs (200 s)) were similar to those of the main findings. Detailed information is presented in the Supplementary Materials. In the 30 TRs window length step analyses, the LB group had significantly decreased dALFF values in the bilateral LING/CAL and left THA, compared with the SC group (Figure S1 and Table S1). Meanwhile, in the 100 TRs window length step analyses, the LB group had substantially decreased dALFF values in the bilateral CAL, compared with the SC group (Figure S2 and Table S1).

## 4. Discussion

Our study is the first of its kind to investigate dynamic spontaneous neural activity changes in LB using dALFF with sliding-window analyses. We showed that individuals with LB displayed significantly lower dALFF values in the bilateral LING/CAL and left THA relative to the SC group. Also, the LB group showed remarkably lower dFC values between the bilateral LING/CAL and the left MFG, as well as between the left THA and the right LING/CER relative to the SC group.

The bilateral LING/CAL is the location of the primary visual cortex in the human brain, which receives visual signals from the visual pathway and transfers them to higher visual cortices. In our previous study, we demonstrated that RP patients had significantly lower ALFF values in the bilateral lingual gyrus/cerebellum posterior lobe relative to the HC group [43]. RP patients also had considerably lower regional homogeneity values in the bilateral lingual gyrus/cerebellum posterior lobe [44]. Hou et al. found that blind patients showed reduced voxel-mirrored homotopic connectivity in the primary visual cortex and visual association cortex, compared with SCs [45]. Qin et al., meanwhile, demonstrated that patients with congenital blindness (CB) and late blindness (LB) had reduced short- and long-range functional connectivity density in the primary visual cortex relative to the SC group [46]. Consistent with these findings, our study revealed that individuals in the LB group had significantly lower dALFF values in the bilateral LING/CAL relative to the SC group. Flexibility in spontaneous neural activity has been associated with behaviorally advantageous changes in brain network dynamics [47]. Thus, our results suggest that reduced flexibility of the brain's activity in the LING/CAL might reflect impaired visual processing in people with LB.

Additionally, we found that LB persons displayed significantly lower dFC values between the bilateral LING/CAL and the left MFG; the frontal lobe was closely linked to higher cognitive function. The MFG is involved in executive attention [48], language [49], and emotion [50]. Previous neuroimaging studies reported robust correlations between the visual cortex and frontal lobe, involving vision-for-action [51] and visuomotor functions [52]. Our results here suggest that lower flexibility of FC between the bilateral LING/CAL and the left MFG might reflect impaired vision-for-action in people with LB.

Remarkably, this study established that individuals with LB had lower dALFF values in the left THA and lower dFC between the left THA and right LING/CER relative to the SC group. The THA is an important subcortical nucleus that transfers various afferents from multiple sensory organs to the primary sensory cortex [53]. Karlen et al. demonstrated that early blindness induces abnormal patterns in thalamocortical and corticocortical connections [54], and Ptito et al. observed abnormalities in the structures of the retinothalamocortical pathway in patients with congenital blindness [55]. Another study revealed that patients with blindness displayed significant alterations in the thalamic microstructure [56]. Consistent with these findings, we speculated that a reduced visual signal input due to blindness might cause the dysfunction of the thalamus. Our results revealed that reduced flexibility of the brain's activity in the left THA might reflect an impaired retinothalamocortical pathway in LB persons.

There are some limitations to this study. First, the selection of the sliding-window length remains a subject of debate. We selected 50 TR as the window length based on the criterion that the minimum length should be more than 1/*f*min. The results of different sliding-window lengths were similar to those of the main results with 50 TR, suggesting that our findings on dALFF were relatively stable. Second, our study used relatively small sample sizes. We intend to use larger sample sizes in the future. Third, the LB group exhibited different ages of blindness onset, which might be associated with heterogeneity in the presentation of LB. Some covariates, including age and gender, meanwhile, were regressed in the statistical analysis to reduce their impact on the accuracy of results. In terms of statistical methods, FDR correction may be used to reduce the bias of results in future studies. Besides, dynamic functional network connectivity method would be used to further reveal the changes in neural mechanisms of blindness in the future study.

## 5. Conclusion

We have shown that individuals with LB exhibited lower temporal variability of dALFF in the visual cortices and thalamus, suggesting lower flexibility in visual thalamocortical activity, which might reflect impaired visual processing in these patients. These findings indicate that abnormal dynamic spontaneous brain activity might be involved in the pathophysiological mechanisms of LB.

## Figures and Tables

**Table 1:** Details of scanned parameters.

Three-dimensional brain volume imaging (3D-BRAVO)		Gradient-echo-planar imaging sequence	
Repetition time/echo time	8.5/3.3	Repetition time/echo time	2,000 ms/25 ms
Slice thickness	1.0 mm	Slice thickness	3.0 mm
Acquisition matrix	256 × 256	Gap	1.2 mm
Field of view	240 × 240 mm2	Acquisition matrix	64 × 64
Flip angle	12°	Flip angle	90°
		Field of view	240 × 240 mm2
		Voxel size	3.6 × 3.6 × 3.6 mm3

**Table 2:** Demographic measurements between the two groups.

	LB group	SC group	*T* values	*P* values
Gender (male/female)	29/12	17/31	11.044	0.001
Age (years)	39.70 ± 12.66	43.23 ± 13.40	-1.267	0.208
Handedness	41 R	48 R	N/A	N/A
Age of onset blindness (years)	22.56 ± 7.13	N/A	N/A	N/A

*χ*2 test for sex (*n*). Independent *t* test for the other normally distributed continuous data (means ± SD). LB: late blindness; SC: sighted controls; N/A: not applicable.

**Table 3:** Significant differences in the dALFF between the two groups.

Condition/brain regions		BA	Peak *T* scores	MNI coordinates			Cluster size (voxels)
				*x*	*y*	*z*	
ROI in B-LING/CAL							
LB<SC	L-MFG	8	-4.2625	-39	12	48	83
ROI in L-THA							
LB<SC	R-LING/CER	—	-4.1493	9	-66	-15	45

The statistical threshold was set at the voxel level with *P* < 0.01 for multiple comparisons using the Gaussian random field theory (voxel-level *P* < 0.01, GRF correction, cluster-level *P* < 0.05). dALFF: dynamic amplitude of low-frequency fluctuation; LB: late blindness; SC: sighted control; LING: lingual gyrus; CAL: calcarine; THA: thalamus; B: bilateral; L: left; GRF: Gaussian random field.

**Table 4:** Significant differences in dFC values between the two groups.

Condition/brain regions		BA	Peak *T* scores	MNI coordinates			Cluster size (voxels)
				*x*	*y*	*z*	
LB<SC	B-LING/CAL	18	-4.5301	0	-72	6	177
LB<SC	L-THA	18	-3.7118	-6	-9	6	105

The statistical threshold was set at the voxel level with *P* < 0.01 for multiple comparisons using the Gaussian random field theory (voxel-level *P* < 0.01, GRF correction, cluster-level *P* < 0.05). dFC: dynamic functional connectivity; BA: Brodmann area; LB: late blindness; SC: sighted control; MNI: Montreal Neurological Institute; GRF: Gaussian random field; LING: lingual gyrus; CAL: calcarine; THA: thalamus; MFG: middle frontal gyrus; CER: cerebelum_6.

## Data Availability

The datasets used and/or analyzed during the current study are available from the corresponding author on reasonable request.

## Supplementary Materials

Supplementary materials Figure S1: comparison of different dALFF values between LB group and SC group (a window size of 30 TRs (60 s), and window shifted by 10 TRs (20 s)). Figure S2: comparison of different dALFF values between LB group and SC group (a window size of 100 TRs (200 s), and window shifted by 10 TRs (20 s)). Table S1: significant differences in the dALFF between two groups.
