# Supplementary material for: Altered Temporal Dynamic Intrinsic Brain Activity in Late Blindness
Source: Biomed Res Int. 2020 Jun 20;2020:1913805. doi: 10.1155/2020/1913805 (PMC7327610; doi:10.1155/2020/1913805)
Supplement: Supplementary materials — Figure S1: comparison of different dALFF values between LB group and SC group (a window size of 30 TRs (60 s), and window shifted by 10 TRs (20 s)). Figure S2: comparison of different dALFF values between LB group and SC group (a window size of 100 TRs (200 s), and window shifted by 10 TRs (20 s)). Table S1: significant differences in the dALFF between two groups. [file 1913805.f1.docx]

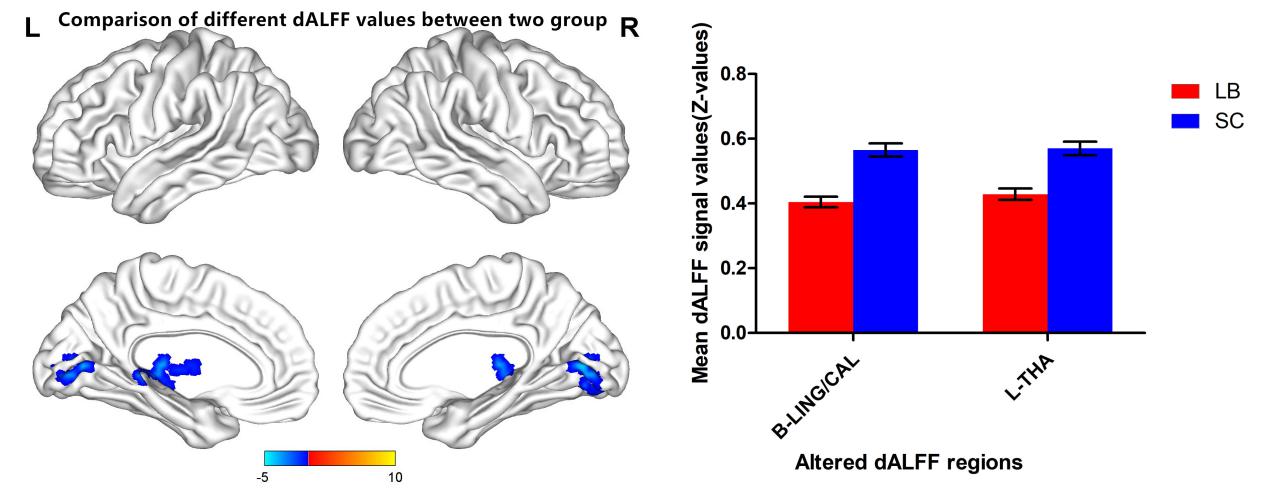


**Figure S1**: Comparison of different dALFF values between LB group and SC group [a window size of 30 TRs (60s), and window shifted by 10 TRs(20s)] .

**Note:**Significant dALFF values differences were observed in the B-LING/CAL, L-THA.The blue areas indicate lower dALFF values.(voxel-level P<0.01, GRF correction, cluster-level P<0.05) (left) The mean values **of altered d**ALFF **values between the LB** **and SC groups.**(right)

**Abbreviations:** dALFF, dynamic amplitude of low-frequency fluctuation; LB, late blindness; SC, sighted controls; GRF,Gaussian random field; LING,lingual gyrus; CAL,calcarine; THA,thalamus; L,left; B, bilateral;


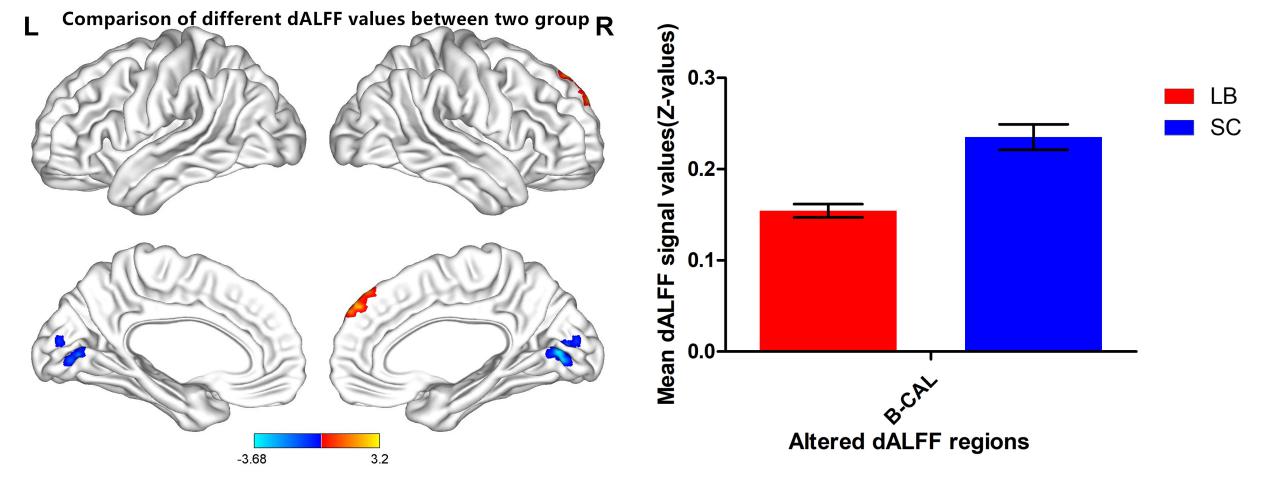


**Figure S2:** Comparison of different dALFF values between LB group and SC group [a window size of 100 TRs (200s), and window shifted by 10 TRs(20s)] .

**Note:**Significant dALFF values differences were observed in the B-CAL.The blue areas indicate lower dALFF values.(voxel-level P<0.01, GRF correction, cluster-level P<0.05) (left) The mean values **of altered d**ALFF **values between the LB** **and SC groups.**(right)

**Abbreviations:** dALFF, dynamic amplitude of low-frequency fluctuation; LB, late blindness; SC, sighted controls; GRF,Gaussian random field; CAL,calcarine; B, bilateral;

| Condition/Brain regions | | BA | Peak  T-scores | MNI coordinates | | | Cluster size (voxels) |
| --- | --- | --- | --- | --- | --- | --- | --- |
|  |  |  |  | x | y | z |  |
| [a window size of 30 TRs (60s), and window shifted by 10 TRs(20s)] | | | | | | | |
| LB<SC | B-LING/CAL | 18 | -4.4868 | 0 | -72 | 3 | 70 |
| LB<SC | L-THA | - | -4.4534 | -6 | -24 | 9 | 20 |
| [a window size of 100TRs (200s), and window shifted by 10 TRs(20s)] | | | | | | | |
| LB<SC | B-CAL | 18 | -3.5568 | 3 | -75 | 3 | 19 |

**Table S1.** Significant differences in the dALFF between two groups

**Note:**The statistical threshold was set at the voxel level with p<0.01 for multiple comparisons using Gaussian random-field theory.(voxel-level P<0.01, GRF correction, cluster-level P<0.05).

**Abbreviations:** dALFF, dynamic amplitude of low-frequency fluctuation; LB, late blindness; SC, sighted control; LING,lingual gyrus; CAL,calcarine; THA,thalamus; B, bilateral;L, left hemisphere; GRF,Gaussian random field;
